# Optimizing the Pharmacological Properties of Discoidal Polymeric Nanoconstructs Against Triple-Negative Breast Cancer Cells

**DOI:** 10.3389/fbioe.2020.00005

**Published:** 2020-02-19

**Authors:** Miguel Ferreira, Ilaria Francesca Rizzuti, Anna Lisa Palange, Maria Grazia Barbato, Valentina Di Francesco, Martina Di Francesco, Paolo Decuzzi

**Affiliations:** ^1^Laboratory of Nanotechnology for Precision Medicine, Fondazione Istituto Italiano di Tecnologia, Genoa, Italy; ^2^Department of Informatics, Bioengineering, Robotics and System Engineering, University of Genoa, Genoa, Italy

**Keywords:** polymeric particles, prodrugs, drug delivery, non-spherical shape, loading and release profiles

## Abstract

Fine-tuning loading and release of therapeutic and imaging agents associated with polymeric matrices is a fundamental step in the preclinical development of novel nanomedicines. Here, 1,000 × 400 nm Discoidal Polymeric Nanoconstructs (DPNs) were realized via a top-down, template-based fabrication approach, mixing together poly(lactic-co-glycolic acid) (PLGA) and poly(ethylene glycol)-diacrylate (PEG-DA) chains in a single polymer paste. Two different loading strategies were tested, namely the “direct loading” and the “absorption loading.” In the first case, the agent was directly mixed with the polymeric paste to realize DPNs whereas, in the second case, DPNs were first lyophilized and then rehydrated upon exposure to a concentrated aqueous solution of the agent. Under these two loading conditions, the encapsulation efficiencies and release profiles of different agents were systematically assessed. Specifically, six agents were realized by conjugating lipid chains (DSPE) or polymeric chains (PEG) to the near-infrared imaging molecule Cy5 (DSPE-Cy5 A and DSPE-Cy5 B); the chemotherapeutic molecules methotrexate (DSPE-MTX and PEG-MTX) and doxorubicin (LA-DOX and DSPE-DOX). Moderately hydrophobic compounds with low molecular weights (MW) returned encapsulation efficiencies as high as 80% for the absorption loading. In general, direct loading was associated with encapsulation efficiencies lower than 1%. The agent hydrophobicity and MW were shown to be critical also in tailoring the release profiles from DPNs. On triple-negative breast cancer cells (MDA-MB-231), absorption loaded DOX-DPNs showed cytotoxic activities comparable to free DOX but slightly delayed in time. Preliminary *in vivo* studies demonstrated the high stability of Cy5-DPNs. Collectively, these results demonstrate that the pharmacological properties of DPNs can be finely optimized by changing the loading strategies (direct vs. absorption) and compound attributes (hydrophobicity and molecular weight).

## Introduction

Nano-based pharmacotherapy deals with the safe, targeted and efficient administration of nanoparticles carrying diverse therapeutic and imaging agents for the treatment and detection of a variety of pathologies, including cancer, cardiovascular, neurological and infectious diseases (Chen et al., 2016). Man-made nanoparticles with a size ranging between a few nanometers and a few micrometers have the potential to optimize the therapeutic efficacy and imaging efficiency of small molecules and biological compounds while minimizing off-target effects (Katsube et al., 2014). Over conventional molecular agents, the encapsulation of therapeutic and imaging compounds into nanoparticles offers several key advantages (Peer et al., 2007; Ahmad et al., 2014). First, nanoparticles enable the systemic administration of agents with poor water solubility, without compromising their chemical structure and preserving their curative and contrast enhancing features. Second, nanoparticles protect delicate biological compounds, such as the various forms of nucleic acids, from a fast enzymatic degradation in blood. Third, the composition, size, shape, and surface properties of the nanoparticles can be tailored, generally, during the fabrication process, thus providing multiple tools to improve delivery to the targeted tissue while minimizing non-specific deposition in healthy, biological districts. Fourth, the release of drugs from nanoparticles can be tuned for an optimal and sustained therapeutic activity (Lu et al., 2008; Slowing et al., 2008; Agasti et al., 2009).

Traditionally, spherical nanoparticles have been extensively used for the treatment and imaging of cancer, relying on the Enhanced Permeability and Retention effect (Maeda et al., 2000). Indeed, sufficiently small nanoparticles (<200 nm) would cross the hyperpermeable tumor blood vessels (enhanced permeability) and accumulate within the tumor parenchyma due to the lack of lymphatic drainage (retention). However, clinical and pre-clinical studies are starting to demonstrate the significant variability of the EPR effect within patients and along the overall development of the disease (Natfji et al., 2017). As such, relying exclusively on the EPR effect to reach the malignant tissue may not always be a successful strategy. More recently, non-spherical particles have been proposed as an alternative drug delivery means to efficiently target the tumor microvasculature and release thereof a variety of therapeutic cargos. These non-spherical nanoparticles could take up different shapes, including rods (Huo et al., 2008), discs (Key et al., 2013), ellipsoids (Desai et al., 2018), and others (Toy et al., 2014). Within this scenario, the authors have previously demonstrated a new nanotechnological platform—the Discoidal Polymeric Nanoconstructs (DPNs). Similarly to red blood cells, DPNs tend to resist sequestration by professional phagocytic cells, such us hepatic Kupffer cells and splenic macrophages (Palomba et al., 2018). This DPN feature results in longer circulation times and increased probability to lodge within the tortuous and low perfused tumor microvasculature (Key et al., 2015).

The Discoidal Polymeric Nanoconstructs are realized with a top-down, template-based approach, where the size, shape, surface properties and mechanical stiffness can be readily and independently modulated (Key et al., 2013; Palange et al., 2017; Palomba et al., 2018). DPNs are made out of poly(lactic-co-glycolic acid) (PLGA) and polyethylene glycol (PEG) chains entangled together to form a hydrogel matrix. Indeed, given the modular fabrication strategy, other synthetic and natural polymeric materials can be used for realizing DPNs, including polycaprolactone, chitosan, hyaluronic acid, and a variety of block-copolymers. PLGA and PEG were selected for their well-known biodegradation and excretion profiles, even in human subjects (Bobo et al., 2016; Park et al., 2019). The geometrical and mechanical configurations of DPNs have been selected to enhance lodging within the malignant tissue by taking advantage of the high vascular tortuosity and low perfusion of tumor capillaries. However, as per any drug delivery system, the accumulation within the tumor microvasculature must be followed by the controlled and sustained release of therapeutic agents directly toward the diseased tissue. As such, in this work, the authors focus their efforts on characterizing and optimizing the pharmacological properties of DPNs.

Here, a systematic analysis of the loading and release for a variety of imaging and therapeutic agents encapsulated into DPNs is conducted. After characterizing the physico-chemical properties of the nanoconstructs, six different compounds are realized by conjugating directly 1,2-Distearoyl-sn-glycero-3-phosphorylethanolamine (DSPE) lipid chains or 1 kDa PEG chains with the near-infrared molecules Cy5 or the chemotherapeutic molecules methotrexate (MTX) and doxorubicin (DOX). The resulting six compounds present different molecular weights and hydrophobicity levels as compared to the original, free molecules. Then, two different loading strategies are introduced named as “direct loading” and “absorption loading.” The six compounds are entrapped within the polymeric matrix of DPNs using both loading strategies, and the encapsulation efficiencies and release profiles are consequently assessed. Finally, the pharmacological and imaging properties of DPNs are documented *in vitro* on triple-negative breast cancer cells (MDA-MB-231) and *in vivo* in healthy mice, respectively.

## Materials and Methodology

### Materials

Silicon wafers (thickness of 525 ± 20 μm, resistivity 20–30 Ω cm, type-orient P/Bar <100>) and the Photoresist AZ 5214 E were purchased from Si-Mat silicon materials (Kaufering, Germany) and Microchemicals (Ulm, Germany), respectively. Hexamethylsilazane (Primer H.M.D.S.) was purchased from Technic. The AZ 726 MIF Developer was purchased from Merk. 1H, 1H, 2H, 2H-Perfluorooctyltrichlorosilane 97% and Methotrexate were purchased from Alfa Aesar. Sylgard 184 kit as polydimethylsiloxane (PDMS) and elastomer were purchased from Dow Coming Corp (Midland, MI, US). Doxorubicin, Poly(vinyl alcohol) (Mw 9,000–10,000, 80% hydrolyzed), Poly(DL-lactide-co-glycolide) acid (PLGA, lactide:glycolide 50:50, Mw 38,000–54,000), Poly(ethylene glycol) dimethacrylate (Mn 750) (PEG dimethacrylate), and 2-Hydroxy-40-(2-hydroxyethoxy)-2-methylpropiophenone (Photo-initiator) were purchased from Sigma (St. Louis, MO, USA). Cyanine5 NHS ester and water-soluble sulfo-Cyanine 5 NHS ester were purchased from Luminoprobe (Hunt Valley, MD, US). All the reagents and solvents were used without further purification.

### Fabrication of the Templates

A master silicon template with circular wells of ~1,000 nm in diameter by 400 nm in thickness was realized. The geometrical pattern of the template was designed using the LASI software. An AZ5214E resist was written via a laser writer system at 405 nm (Heidelberg Instruments DWL66FS). The primer and the resist were deposited over the silicon wafer by spin coating (Sawatec) (10 s at 500 rpm and 60 s at 4,000 rpm) resulting in a final thickness of about 1,400 nm. This coating was baked on a hot plate at 110°C for 1 min and then exposed to the Direct Laser Writer working with a defocusing value of 1,700 nm and laser intensity of 0.75 mW. The substrate was developed for 4 min. The pattern was transferred to the underlying silicon substrate by deep reactive ion etching with SF_6_/O_2_ plasma for 60 s (Sentech SI500 Instrument GmbH RIE). The resist was removed in a piranha solution for 5 min (ratio 4:1 between sulfuric acid and hydroxide peroxide). The resulting silicon master template was imaged with Scanning Electron Microscopy (Helios Nanolab 650 Dual Beam, Fei company). A silanization step (200 μl) was also included by placing the substrate in a vacuum chamber under a nitrogen atmosphere for 1 h. Once the silicon master template was obtained, the following step required the realization of a polydimethylsiloxane (PDMS) replica. The silicon master template was fixed on a Petri dish (*d* = 10 cm). A solution of PDMS and curing agent was realized with a ratio 10:1. This solution was cast over the Si wafer to produce a second template presenting cylindrical pillars, which resembled the same size and shape of the wells in the original master Si template. The mixture was polymerized at 60° for 3 h. This process was repeated seven times to generate seven different PDMS templates. Then, ~30 mg of PDMS (10:1) were poured over these replicas to form a unique structure with the size of a Petri dish (*d* = 15 cm). The final step required the realization of a sacrificial template of poly(vinyl) alcohol (PVA). A 5% PVA solution was deposited over the PDMS replica template to generate a third template. This presented circular wells just like the original master Si template. Upon complete drying in an oven for 3 h at 60°C, the PVA solution became a thin film and was peeled off the PDMS template.

### Fabrication of Discoidal Polymeric Nanoconstructs

For the fabrication of DPNs, 30 mg of poly(lactic-co-glycolic acid) (PLGA) were dissolved in chloroform (CHCl_3_) and mixed with 6 mg of polyethylene glycol (PEG) diacrylate and 10 μg of 2-Hydroxy-40-(2-hydroxyethoxy)-2-methylpropiophenone (photoinitiator). Thirty microgram of DSPE-Cy5 were also added to provide sufficient fluorescent optical contrast. The resulting polymeric paste was carefully deposited in the wells of the sacrificial PVA template and exposed to UV-light for polymerization. The resulting PVA film, loaded with the above polymeric paste, was immersed in water and left to stir for 3 h. This step led to the dissolution of the PVA and the release of DPNs in the aqueous solution. DPNs were eventually collected *via* centrifugation and membrane filtration to remove residual fragments of PVA.

### Loading of the Discoidal Polymeric Nanoconstructs

Two loading protocols were developed, namely “direct loading” and “absorption loading.” In “direct loading,” the imaging and/or therapeutic agents of interest were dispersed within the original polymeric paste and directly distributed in the wells of the PVA template. In “absorption loading,” the collected DPNs were lyophilized to form a powder. This was eventually dispersed in an aqueous solution carrying the imaging and/or therapeutic agents of interest. Re-hydration led to the rapid absorption of the molecules within the hydrogel structure of DPNs. The concentration of DPNs, imaging agents and therapeutic molecules, the volume of the aqueous solution was systematically varied to identify optimal loading conditions.

### Prodrug Synthesis

#### DSPE-Cyanine-5

DSPE-Cy5-A and DSPE-Cy5-B were synthesized as reported by Lee and coworkers with some modifications (Lee et al., 2017). 15 mg of DSPE-NH_2_ were dissolved in 3 mL of dichloromethane (DCM) and 1.5 mL of MeOH. 0.98 eq of Cyanine-5 NHS ester was dissolved in 200 ml of dimethylformamide (DMF) and added to the previous solution. A catalytic amount of triethylamine (TEA) was added to the reaction and left to stir for 16 h. The resulting product was precipitated with cold diethyl ether, then washed three times with cold diethyl ether obtaining the final compound with a yield of 90%.

#### DSPE-Methotrexate

Fifteen milligram of DSPE-NH_2_ were dissolved in 3 mL of dichloromethane (DCM) and 1.5 mL of MeOH. 0.98 eq of Methotrexate ester was dissolved in 200 ml of dimethylformamide (DMF) and added to the previous solution. A catalytic amount of triethylamine (TEA) was added to the reaction and was left to stir for 16 h. The intended product was precipitated with cold diethyl ether, then washed three times with cold diethyl ether getting the final product with a yield of 90%.

#### PEG-Methotrexate

Twenty milligram of PEG-NH_2_ (1,000 Da) were dissolved in 3 mL of dichloromethane (DCM) and 1.5 mL of MeOH. 0.98 eq of Methotrexate was dissolved in 200 ml of dimethylformamide (DMF) and added to the previous solution. A catalytic amount of triethylamine (TEA) was added to the reaction and was left to stir for 16 h. The intended product was precipitated with cold diethyl ether, then washed three times with cold diethyl ether getting the final product with a yield of 90%.

#### Linoleic Acid-Doxorubicin

LA-DOX was synthesized as reported by Fernandes et al. (2016) with some modifications. Briefly, an aqueous solution of Doxorubicin hydrochloride (5 mg/mL) was neutralized with a solution of sodium bicarbonate solution (50 mg/mL). A linoleic acid solution (50 mg/mL) in ethanol was incubated with EDC/NHS (molar ratio of 3:1) for 1 h under magnetic rotation at room temperature. The obtained solution was added drop by drop to the above mixture while stirring. After 12 h, the mixture was centrifuged (127,000 rpm, 30 min). The red pellet was washed with distilled water three times, removing the unreacted water-soluble Doxorubicin. Finally, the red pellet was collected and dried under vacuum.

#### DSPE-Doxorubicin

An aqueous solution of Doxorubicin hydrochloride (5 mg/mL) was neutralized with a solution of sodium bicarbonate solution (50 mg/mL). A DSPE-succinic-acid (50 mg/mL) in ethanol was incubated with EDC/NHS (molar ratio of 3:1) for 1 h under magnetic rotation at room temperature. The obtained solution was added drop by drop to the above mixture while stirring. After 12 h, the mixture was centrifuged (127,000 rpm, 30 min). The red pellet was washed with distilled water three times, removing the unreacted water-soluble Doxorubicin. Finally, the red pellet was collected and dried under vacuum.

### Particle Size and Shape Characterization

The size and shape of all silicon, PDMS, and PVA templates were characterized via Scanning Electron Microscope (Helios Nanolab 650). Ultra-high resolution SEM images were acquired at high vacuum conditions after 10 nm aurum coating using a Q150T ES sputter-coater (Quorum). DPNs still embedded within the PVA templates were observed using an A1 confocal microscope (Nikon) equipped with 63 × oil immersion objective. For EM characterization, a DPNs solution was dried on a carbon-copper grid and coated with 10–20 nm of carbon before Transmission Electron Microscope imaging (JEOL JEM 1011 TEM working at 100 KV). The ζ-potential was calculated using DLS (Malvern, UK).

### Loading and Release Studies

To calculate the amount of drug inside DNPs, samples were lyophilized and dissolved in acetonitrile (ACN). All samples were analyzed by HPLC at 240 nm UV absorbance (Agilent 1260 Infinity, Germany). The encapsulation efficiency was defined as the percentage weight ratio between the drug amount loaded inside DPNs at the end of their preparation and the initial input amount of the drug. For the release studies, 200 μL of DPN solution was poured into Slide-A-Lyzer MINI dialysis microtube with a molecular cut off of 10 kDa (Thermo Scientific) and dialyzed against 4 L of H_2_O at 37°C. For each time point, in triplicate, DPNs were collected and destroyed with ACN to release the molecule of interest. Samples were analyze depending on an HPLC (Agilent 1260 Infinity, Germany) at 340 nm UV absorbance for MTX prodrugs, and 490 nm and 646 for LA-DOX and Cy5, respectively.

### Cell Culture and Viability

The human Triple-Negative Breast Cancer MDA-MB231 cell line was obtained from the American Type Culture Collection (ATCC). Cells were cultured in Eagle's minimal essential medium (EMEM) (ATCC, USA) containing 10% FBS (Gibco, Thermo Fisher Scientific, USA), 1% penicillin/streptomycin (Sigma-Aldrich, USA), under a humid atmosphere (37°C, 5% CO_2_, 95% air). Upon reaching appropriate confluence, cells were passed. Cell viability was determined via an MTT assay, which detects the reduction of MTT [3-(4,5-dimethylthiazolyl)-2,5-diphenyltetrazolium bromide] (Sigma-Aldrich, USA) by mitochondrial dehydrogenase to blue formazan product. This reflects the normal function of mitochondria and, hence, the measurement of cytotoxicity and cell viability. Briefly, 2 × 10^5^ cells/well were seeded in 96-well plates and incubated at 37°C, 5% CO_2_, for 24 h. Next, the medium was replaced with EMEM containing the corresponding concentrations of Doxorubicin (DOX), doxorubicin pro-drug [LA-DOX, DSPE-DOX, (LA-DOX) DPNs and (DSPE-DOX) DPNs)] (0.1–50 μM), Methotrexate (MTX) and Methotrexate prodrugs [PEG-MTX, DSPE-MTX (0–100 μM) and (DSPE-MTX) DPNs (0.01–0.5 μM)]. After 24, 48, 72, and 96 h of incubation, the MTT solution (5.0 mg/mL PBS) was added to each well and incubated at 37°C for 4 h. The resulting formazan crystals were dissolved by adding ethanol (200 μL/well), and the absorbance was read at 570 nm using a microplate reader (Tecan, CH). Controls (i.e., cells that had received no drug) were normalized to 100%, and readings from treated cells were expressed as the percentage of viability inhibition. Five replicates were considered for each data point.

### Confocal Fluorescent Microscopy Imaging

Cellular uptake and intracellular localization were observed in MDA-MB-231 cells for DOX, LA-DOX, and DPNs. Briefly, 2.0 × 10^4^ cells were seeded into each well of a Nunc Lab-Tek II chamber slide system (Thermo Fisher Scientific, USA) with standard culturing conditions (37°C, 5% O_2_). After 24 h, the medium was removed, and cells were washed in PBS for 5 min (Thermo Fisher Scientific, USA). Cell fixation was performed using a 4% solution of paraformaldehyde (Santa Cruz Biotechnology, USA) for 5 min. Actin was stained with Alexa Fluor 488 Phalloidin (green color) (Thermo Fisher Scientific, USA), and nuclei were stained with DAPI (blue color) (Thermo Fisher Scientific, USA). Doxorubicin and DSPE-DOX DPNs were visualized in red.

### *In-vivo* Studies

DPNs loaded with DSPE-Cy5-B were injected into week old C57B/6J female mice (Charles River). Animals were grouped in ventilated cages and able to freely access to food and water. They were maintained under controlled conditions: temperature (21 ± 2°C), humidity (50 ± 10%) and light (10 and 14 hours of light and dark respectively). The accumulation of systemically injected DSPE-Cy5-B DPNs within the different organs was assessed longitudinally by using the whole animal, near infra-red fluorescent (NIRF) imaging at 0.5, 1, 2, 6, 24 and 48 h. Also, at 48 h post-injection, the major organs were imaging ex-vivo.

## Results and Discussion

### Fabrication and Physico-Chemical Characterization of the Discoidal Polymeric Nanoconstructs (DPNs)

**Figure 1:**
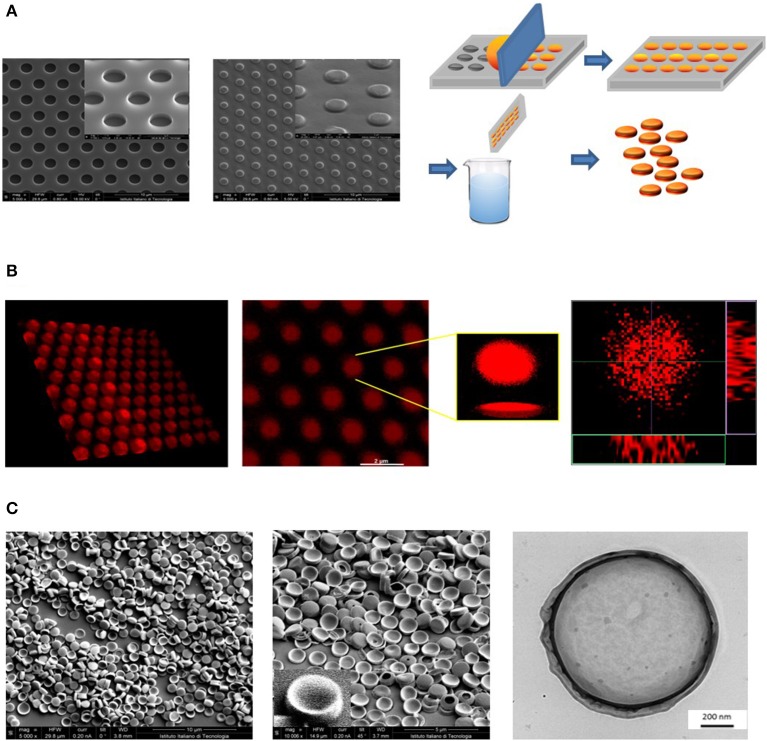
Geometrical characterization of Discoidal Polymeric Nanoconstructs (DPNs). **(A)** Scanning electron microscope (SEM) images of the silicon templates (left); SEM images of the PDMS templates (center); schematic of the DPN fabrication steps, including loading of the PLGA/PEG polymer mixture, polymerization, sacrificial template dissolution and DPN collection (right). **(B)** Confocal microscopy images of a PVA template filled with DOX-loaded DPNs (left) and individual, 3D reconstructed DOX-loaded DPNs (right). **(C)** SEM (left) and Transmission Electron Microscopy (right) images of DPNs.

A versatile fabrication strategy was used to realize polymeric nanoconstructs with controlled geometrical and mechanical features, including the size, shape, surface properties, and mechanical stiffness–*the 4S parameters*. These features can be independently tuned in the fabrication process to affect the therapeutic and imaging performances, as shown by the authors and other investigators (Key et al., 2013; Palomba et al., 2018). The first step in DPNs fabrication is the realization of a master silicon template through a Direct Laser Writer lithographic process. The silicon template appears as a matrix of wells reproducing the geometry of DPNs (Figure 1A, left inset). Then, the silicon master template is used as a mold to realize a PDMS replica. The PDMS replica exhibits arrays of pillars with the same geometry of the wells in the silicon template. Finally, a sacrificial PVA template is obtained by replicating the PDMS template. DPNs are made out of a mixture of poly(lactic-co-glycolic acid) (PLGA), polyethylene glycol diacrylate (PEG-DA), photoinitiator (PI), and the therapeutic or imaging agent of interest. For the preparation of DPNs, the polymer mixture of PLGA, PEG-DA, and PI is directly applied and spread over the PVA template to fill the multiple wells (Figure 1A, right inset). This loaded template is then exposed to UV-light for the polymerization of PEG-DA chains. Eventually, the PVA film is immersed in an aqueous solution and dissolved after stirring (Figure 1A, right inset) to release the DPNs. The final steps, including centrifugation and filtration, are used to remove residual PVA fragments. Figure 1B presents confocal fluorescent images of a PVA template loaded with a mixture of PLGA/PEG and LA-DOX. A uniform distribution of “red dots” within the structure can be appreciated. These “red dots” correspond to the DPNs before the dissolution of the PVA template and are loaded quite uniformly with LA-DOX, returning the reddish color. Finally, electron microscopy images (Figure 1C) show the actual geometry of DPNs that exhibits an average diameter of ~1,250 ± 19.36 nm and a height of 469.14 ± 27.5 nm.

### Loading Therapeutic and Imaging Agents Into Discoidal Polymeric Nanoconstructs (DPNs)

**Figure 2:**
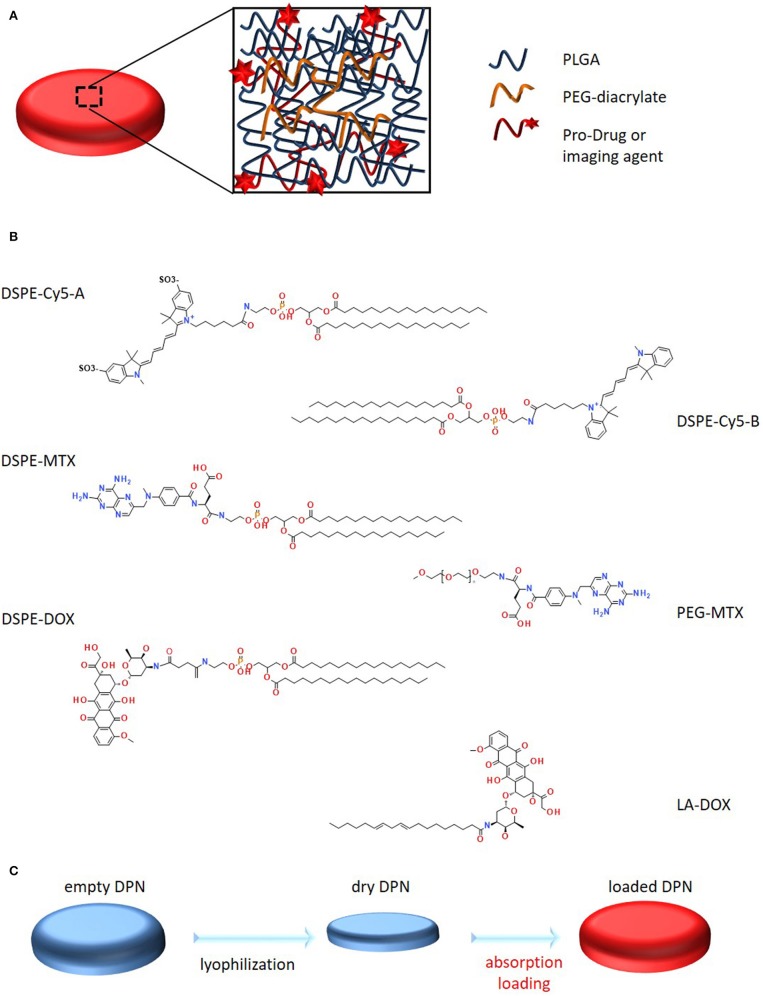
Schematic representation of Discoidal Polymeric Nanoconstructs and prodrugs. **(A)** Schematic representation of DPNs, highlighting their porous structure and PLGA-PEG chain entanglement. **(B)** Structural representation of the six synthesized pro-drugs. **(C)** Schematic representation of the “absorption loading” method for encapsulating prodrugs into DPNs.

The dispersion of functional molecules, such as therapeutic and imaging agents, within the polymeric structure of DPNs (Figure 2A) was achieved following two different strategies: a “direct method,” where the agent is dissolved in the organic solvent and directly mixed with the PLGA, PEG and PI paste prior to deposition over the PVA template; an “absorption method,” where the agent is dispersed in a higher concentrated water solution, which is then exposed to the lyophilized powder of DPNs. In direct loading, the agent needs to be soluble and stable in an organic solvent whereas, for the absorption method, the agent needs to be soluble in water at high concentrations without forming micellar structures. As such the proposed different loading methods can be applied to different functional agents, even sequentially.

DSPE-Cy5-A was the first compound to be loaded into the DPNs (Figure 2B). The Cyanine 5 (Cy5) molecule is a well-known and extensively used near infra-red molecule that can be easily detected *in vitro*, using a conventional fluorescent microscope, and *in vivo*, using a whole animal fluorescent imaging system. DSPE-Cy5-A was synthesized by reacting 1,2-distearoyl-sn-glycero-3-phosphoethanolamine-N-amino (DSPE-NH2) with Cy5 pre-activated with N-Hydroxysuccinimide (NHS) on the terminal carboxylic acid. The compound DSPE-Cy5-A resulted in being less hydrophobic than free Cy5-A. A more hydrophobic Cy5, which lacks the sulfoxide groups, increased, even more, the hydrophobicity of the compound and was used to prepare DSPE-Cy5-B (Figure 2B). The same protocol was used for the synthesis. Then, methotrexate (MTX) and doxorubicin (DOX) were considered. These are two chemotherapeutic molecules with different solubility properties in physiological solutions. MTX is hydrophobic whereas DOX is hydrophilic. A similar protocol as per the Cy5 was used to prepare the prodrug DSPE-MTX (Figure 2B). MTX was pre-activated with a mixture of DCC and NHS, before conjugation with DSPE-NH_2_. With MTX, the hydrophobicity of the resulting compound was modulated by changing the lipid chain DSPE with PEG 1 kDa (Figure 2B), to increase the hydrophilicity of the resulting MTX prodrug. The synthesis protocol was the same as described above. Then, Doxorubicin (DOX) was considered, which is currently the most frequently used treatment for triple-negative breast cancer (Denard et al., 2018). In this case, the protocol to generate the DOX prodrug was modified, in that DOX has no carboxylic acid group available, but rather an amine group. Therefore, first, the hydrochloridric form of DOX was neutralized with a solution of sodium bicarbonate. Then, linoleic acid was pre-activated with EDC and NHS, and added to the solution. Given the low water solubility of the resulting compound, it was collected via precipitation. The same procedure was used for the preparation of the DSPE-DOX (Figure 2B), replacing the linoleic acid with the DSPE-succinic acid. After preparing all these compounds and prodrugs, the loading steps were performed.

“Direct loading” is the most straightforward procedure. The main requirement for the agent to be “direct loaded” is its solubility and stability in organic solvents, like dichloromethane (DCM), chloroform (CHCl_3_), or acetonitrile (ACN). The compound is directly mixed with the original polymeric paste forming the DPNs and applied over the PVA template. A drawback of this straightforward loading procedure is the low encapsulation efficiency (EE), which is due to the yielding in the preparation of DPNs and the hydrophilicity-hydrophobicity ratio of the loaded agent. As per the yielding, it should be noted that a portion of the loaded agent and polymeric paste are wasted during the deposition onto the PVA wells. As per the hydrophilicity-hydrophobicity ratio, it should be remarked that more hydrophilic compounds (lower LogP) tend to rapidly escape the DPN polymeric matrix during the purification and collection steps that occur in water.

“Absorption loading” consists in exposing a concentrated aqueous solution of the agent to lyophilized DPNs (Figure 2C). During the rapid re-hydration phase, DPNs avidly recall within their polymeric structure water, which was enriched with the molecules of the agent to be loaded. A fine balance between compound hydrophilicity and hydrophobicity is required for this loading strategy. Note that, after absorption, the higher molecular-weight hydrophobic prodrugs would more easily stay entrapped within the core of the DPNs as compared to their free counterparts, thus limiting their interaction with the aqueous environment.

### Optimizing the “Absorption Loading” Into Discoidal Polymeric Nanoconstructs (DPNs)

**Figure 3:**
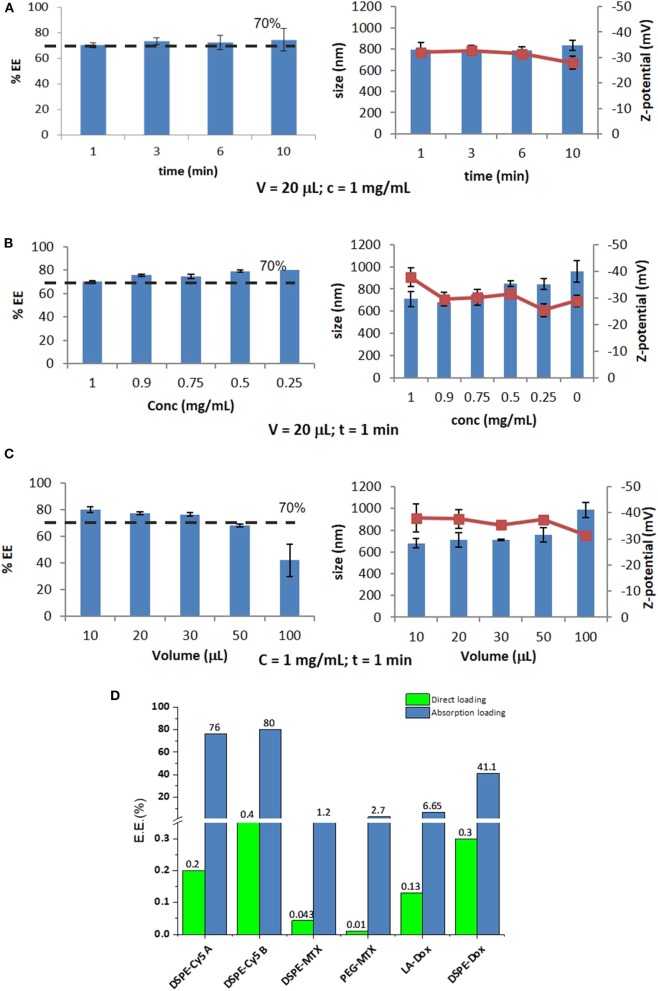
Optimization of Absorption Loading and Encapsulation Efficiency into Discoidal Polymeric Nanoconstructs. Variation of the encapsulation efficiency and DPN size and surface ζ-potential as a function of **(A)** the rehydration time; **(B)** the pro-drug mass; and **(C)** the volume of the rehydration solution. **(D)** Encapsulation efficiencies for the six different pro-drugs and two different loading methods (“direct method”—green bars and “absorption method”—blue bars).

Based on the above reasoning, the encapsulation efficiency (EE) into DPNs would vary for each prodrug and loading strategy. In the “direct loading,” EE can be optimized by improving the filling of the wells in the PVA template and limiting the losses of polymeric paste out of the wells during the deposition phase. This optimization process is out of the scope of this work. As per the “absorption loading,” three independent parameters can be systematically modified to modulate EE. These parameters are: the exposure time of the lyophilized DPNs to the aqueous solution (rehydration time); the mass of prodrug in the aqueous solution; the volume of the aqueous solution. For studying the contribution of the rehydration time, lyophilized DPNs were exposed to a 20 μL concentrated solution of 1 mg/mL DSPE-Cy5-B for 1, 3, 6, and 10 min. After exposure, water was added to the mixture followed by a centrifugation step to remove the non-absorbed DSPE-Cy5-B molecules. This was quantified via UV-Vis spectroscopy. The results showed that Cy5 loading stayed around 75% and no variation with the exposure time was observed (Figure 3A, left column). Also, DPNs size and ζ-potential did not change during the process demonstrating that DPNs did not suffer any significant damage induced by lyophilization and rehydration (Figure 3A, right column). Based on this data, 1 min of rehydration time was considered to be sufficient to load a significant amount of DSPE-Cy5-B. “Absorption Loading” is also affected by the volume of the aqueous solution and the concentration of the therapeutic agent. Therefore, after fixing the volume of DSPE-Cy5-B solution (20 μL) and the exposure time (1 min), the agent concentration was reduced from 1 to 0.9, 0.75, 0.5, and 0.25 mg/mL. Even in this case, the compound concentration did not significantly affect the loaded amount of DSPE-Cy5-B, which stayed around 75% (Figure 3B, left column). A modest change in DPNs size was observed with a decreasing concentration of the compound (Figure 3B, right column). At low concentrations, DPNs acquired a larger size (860 nm, at 0.25 mg/ml) compared to the higher concentrations (720 nm at 1 mg/mL). This could be related to the intercalation of the compound within the DPN polymeric matrix that would pull it toward the core resulting in a slightly smaller and more compact structure. The last independent parameter to be analyzed was the volume of rehydration. In this case, the compound concentration (1 mg/ml of DSPE-Cy5-B) and the exposure time (1 min) were fixed, whereas the volume varied between 10, 20, 30, 50, and 100 μL. Results show that with a volume increase, the loading of the DSPE-Cy5-B decreases (Figure 3C, left column). Indeed, lower volumes mean more DSPE-Cy5-B located in the vicinity of the DPN surface, thus leading to higher loading upon absorption. DPN size and ζ-potential were not affected by the volume variation (Figure 3C, right column).

Summing all this data up, it can be concluded that optimal loading via absorption is achieved by minimizing rehydration volumes and maximizing the mass of the compound. Importantly, the compound should be sufficiently hydrophilic to be dispersed in water and moderately hydrophobic to stay within the DPN matrix upon exposure to the aqueous environment. Thus, for this loading strategy, it is crucial to realize prodrugs with the right hydrophilic-hydrophobic ratio and molecular weight. The bar chart in Figure 3D summarizes the results obtained in terms of EE for the different loading strategies and tested compounds. The DSPE-Cy5 A and B present high EE under the absorption loading strategy, reaching values as high as ~80%. Because of the DSPE derivation, these compounds are slightly less hydrophilic and have a larger molecular weight as compared to the original free molecules (Cy5 A and B). These two features favor DSPE-Cy5 loading into DPNs during the absorption phase and limit their release during the purification steps and the *in vivo* application. This ideal combination of hydrophobicity/hydrophilicity ratio and the proper molecular weight is not achieved with the MTX prodrugs. The DSPE-MTX is significantly hydrophobic, with a LogP of 13.84, and cannot be efficiently resuspended in water returning an EE = 1.2%. On the other hand, the 1 kDa PEG-MTX has a LogP of −3.31, which allows efficient resuspension in water. However, the large molecular weight of the resulting compound opposes absorption during the DPN rehydration, thus returning a moderate EE of ~3%. The beneficial effect of identifying the right hydrophobicity/hydrophilicity ratio is again demonstrated in the case of DOX. LA-DOX is significantly hydrophobic with a LogP of 6.59. This leads to an EE of ~7%, which incidentally is higher than that obtained for the more hydrophobic DSPE-MTX. Notably, DSPE-DOX has a LogP of −14.42, returning an EE of ~40%. Figure 3D also shows how direct loading, in the current configuration, is far less efficient than absorption loading. This data confirms that the hydrophobicity/hydrophilicity ratio and molecular weight of the prodrug are critical features to optimize loading.

### Release Profiles of Prodrugs From Discoidal Polymeric Nanoconstructs (DPNs)

**Figure 4:**
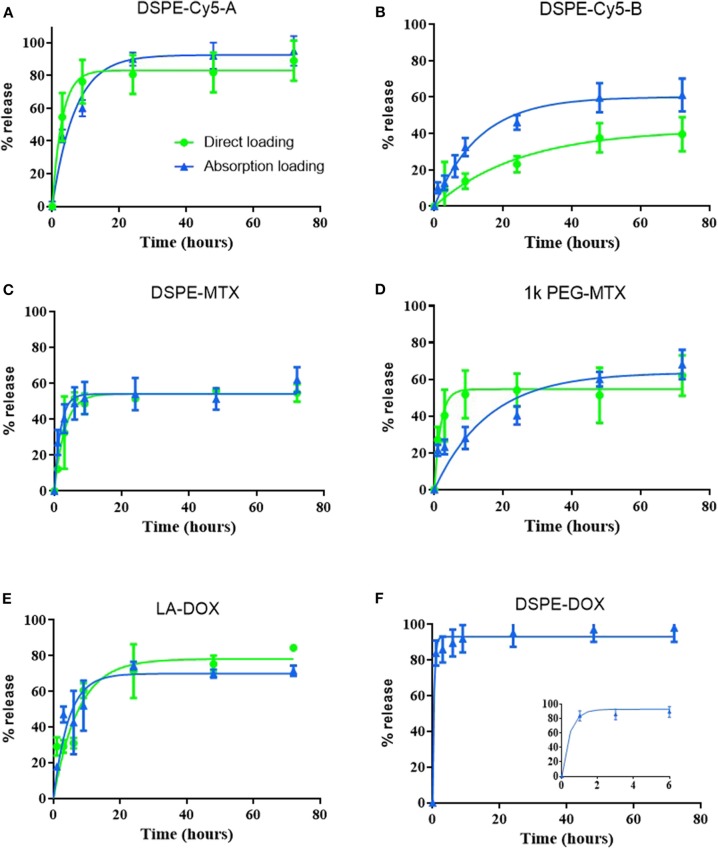
Release profiles of the prodrugs from Discoidal Polymeric Nanoconstructs. Release profiles for **(A)** DSPE-Cy5-A; **(B)** DSPE-Cy5-B; **(C)** DSPE-MTX; **(D)** PEG-MTX; **(E)** LA-DOX; and **(F)** DSPE-DOX loaded into DPNs via the “direct method” (• green line) and the “absorption method” (▴ blue line).

Release studies were conducted for all loaded prodrugs under the sink condition by exposing a known amount of DPNs to a 4 L aqueous solution. Starting with DSPE-Cy5-A (Figure 4A), an initial burst release was observed over the first 10 h with ~70 and 80% of the agent leaking out of DPNs for the absorption and direct methods, respectively. At 72 h, the DSPE-Cy5-A loaded via the absorption method was fully released out of DPNs as compared to a 90% release for the direct method. Indeed, the DSPE-Cy5-A suffers of a high water solubility, which would justify the observed rapid release profile. Moreover, in the absorption method, most of the DSPE-Cy5-A molecules are likely trapped near the DPN surface, which indeed facilitates their release in the surrounding aqueous environment. On the other hand, the direct loading of DSPE-Cy5-A favors its uniform distribution within the DPN polymeric matrix, thus slowing down the release rate. A different behavior is observed for the more hydrophobic compound DSPE-Cy5-B (Figure 4B). Over the first 10 h, only 35 and 12% of the loaded DSPE-Cy5-B are released out of DPNs for the absorption and direct methods, respectively. At 72 h, these two values become 60 and 35%, respectively. The slower release of DSPE Cy5-B as compared to DSPE Cy5-A should be ascribed entirely to the different hydrophobic/hydrophilic ratio, which is in favor of the Cy5-B compound. Notably, the direct method is still characterized by a lower release rate, likely because of the uniform distribution of the compound within the DPN polymer matrix. The release profiles for DSPE-MTX and PEG-MTX are presented in Figures 4C,D, respectively. Due to the different hydrophobicity level, these compounds present a different release behavior as opposed to DSPE-Cy5. The release of DSPE-MTX was characterized by an initial burst over the first 6 h with 50% of the drug being released for both loading methods. Interestingly, the release of DSPE-MTX was observed to plateau around 60% at about 24 h. A similar observation applies for PEG-MTX but only for the direct method loading. For the absorption loaded PEG-MTX, the compound leaked out of the DPN matrix in a sustained fashion over the whole period of observation. Note that DSPE-MTX is the most hydrophobic compound realized in this work and, as such, upon direct loading into DPNs, it prefers to stay associated within the PLGA/PEG matrix. This would explain the plateau observed in Figure 4C. Interestingly, the value of the plateau is similar for both direct loaded DSPE-MTX and 1 kDa PEG-MTX, thus confirming the importance of modulating the compound hydrophobicity. On the other hand, the absorption loaded 1 kDa PEG-MTX, which is mostly confined to the DPN surface given its high molecular weight, escapes the particle matrix just as DSPE-Cy5-B. The LA-DOX release is documented in Figure 4E. The release profiles for the direct and absorption loaded compound are quite similar to a moderate burst release within the first 24 h. Indeed, this initial burst was lower than for the MTX as, over the first 10 h, 55 and 50% of the LA-DOX was released via the absorption method and direct method, respectively. Then, a total of 70 and 65% LA-DOX was released at 24 h, respectively. The DSPE-DOX (Figure 4F) was only loaded through the absorption method, given its hydrophilicity. After the first hour, 80% of DSPE-DOX was already released out of DPNs. Note that DSPE-DOX is more soluble in water than LA-DOX, and this would explain the faster release rates documented for the first compound as compared to the second compound. This data confirms that the hydrophobicity/hydrophilicity ratio and molecular weight of the prodrugs play a critical role also in optimizing the release profiles.

### Cell Viability Studies for the Prodrugs and Discoidal Polymeric Nanoconstructs

**Figure 5:**
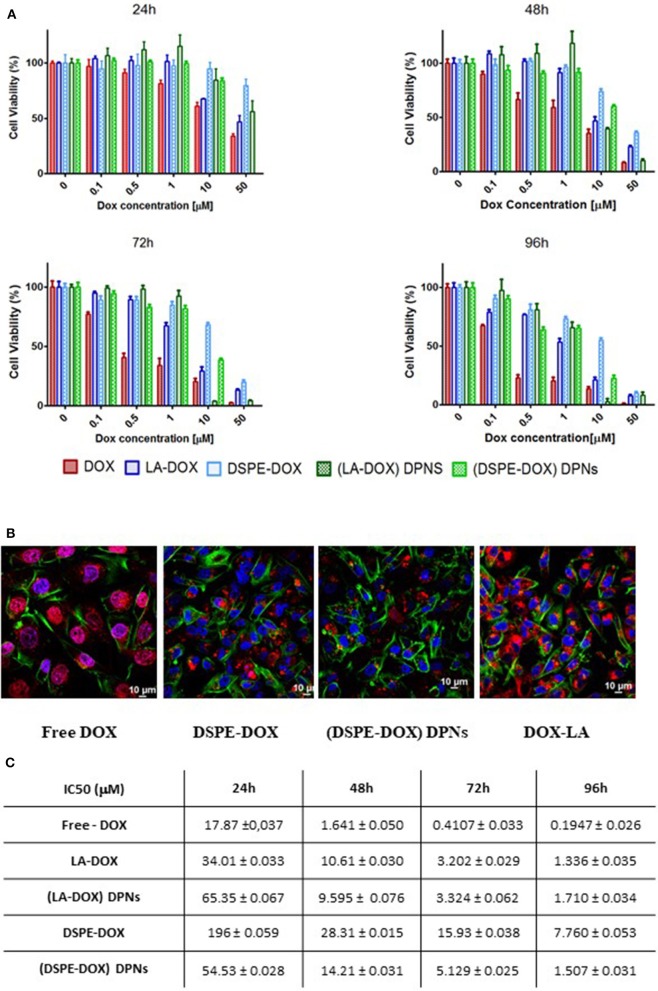
Cytotoxic activity of DOX prodrugs and DOX-loaded Discoidal Polymeric Nanoconstructs. **(A)** Cell viability analysis of free-DOX, DSPE-DOX, DSPE-DOX DPNs, and LA-DOX at 24, 48, 72, and 96 h of incubation time on triple-negative breast cancer cells (MDA-MB-231). **(B)** Representative confocal microscopy images of free-DOX, DSPE-DOX, DSPE-DOX DPNs, and LA-DOX in MDA-MB-231 at 96 h of incubation time. **(C)** Summarizing table listing the IC50 values for free-DOX, DSPE-DOX, DSPE-DOX DPNs, and LA-DOX onto MDA-MB-231 at 24, 48, 72, and 96 h of incubation time.

Using the MTT cell proliferation assay, the cytotoxic activity of prodrugs and DPNs was tested on MDA-MB-231 breast cancer cells at 24, 48, 72 and 96 h (Supplementary Figures 1A–E). In line with the existing literature, free MTX and PEG-MTX manifested a modest cytotoxicity (Wei et al., 2019). On the other hand, DSPE-MTX presented a much lower IC50, ranging between a few tens of nanomolars to a few micromolars (Supplementary Figures 1A–C). However, the intrinsically low cytotoxicity of the drug together with the moderate encapsulation efficiency in DPNs return an insufficiently high cell killing activity for MTX-loaded DPNs (Supplementary Figure 1D). Consequently, cell viability analyses were mostly focused on DOX prodrugs and DOX-DPNs. In general, LA-DOX prodrug treatments were associated with a delayed response as compared to the free DOX. Specifically, at 24 h, the IC50 increased from 17.8 μM for the free DOX to 34.01 μM for LA-DOX (Figures 5A–C). This trend was documented for all tested conditions and should be ascribed to the hydrolyzation of the amide bond between DOX and oleic acid, which indeed requires times to progress. Importantly, LA-DOX DPNs were still associated with similar values of cell-killing potential. Only at 24 h, the IC50 values associated with LA-DOX DPNs were higher than for free LA-DOX (Figure 5C). As per DSPE-DOX, the loss in activity was even higher. At 24 h, the IC50 increased from 17.8 μM for free DOX to 196 μM for DSPE-DOX (Figure 5C). Even at 96 h, there was a reduction in IC50 of about 50 times as compared to DOX and 5 times as compared to LA-DOX. However, the IC50 values for the DSPE-DOX and LA-DOX loaded DPNs were documented to be quite similar at 48, 72, and 96 h of incubation time (Figure 5C). Similar results were found by Sui and co-workers for free DOX and its prodrug (DOX-PEG) (Gou et al., 2013).

In order to characterize drug uptake in triple-negative breast cancer, the MDA-MB-231 cells were incubated with free DOX, prodrugs (DSPE-DOX and LA-DOX) and prodrug-loaded DPNs for 24, 48, 72, and 96 h. Representative confocal fluorescent images are provided in Figure 5B and Supplementary Figures 2–5. Both free DOX and free LA-DOX were observed to rapidly enter the cell and distribute within the cytosol around the cell nuclei. This process was also observed for the DSPE-DOX and the DPNs loaded with DSPE-DOX and LA-DOX. However, in these latter three cases, cell internalization was significantly delayed in time. In the case of DSPE-DOX, this delay should be ascribed to the lower efficiency of this prodrug in permeating through the cell membrane, as opposed to DOX and LA-DOX. In the case of prodrugs-loaded DPNs, the delay is due to the fact that, first, the prodrug would beed to be released from the nanoconstructs into the surrounding solution, and, after that, the prodrug would permeate across the cell membrane and eventually reach the cytosol (Supplementary Figures 2–5).

### Pre-clinical Biodistribution Studies for the Discoidal Polymeric Nanoconstructs (DPNs)

**Figure 6:**
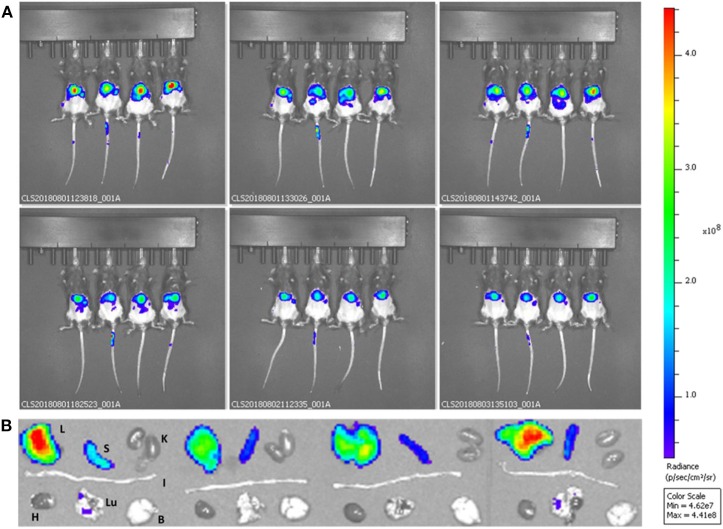
Preliminary biodistribution studies of Discoidal Polymeric Nanoconstructs in healthy wild type C57BL/6 mice. **(A)** Whole animal optical fluorescent images at different time points post-DSPE-Cy5-DPN tail vein injection. **(B)** *Ex vivo* optical fluorescent images of harvested organs—Liver (L), Spleen (S), Kidneys (K), Intestine (I), Heart (H), Lungs (LU), and Brain (B) for DSPE-Cy5-DPNs (*n* = 4).

DPNs directly loaded with DSPE-Cy5-B were administered into immunocompetent black mice via tail vein injection. The accumulation of DSPE-Cy5-B DPNs in the different organs was assessed longitudinally by using a whole animal, near infra-red fluorescent (NIRF) imaging system (Figure 6A). Representative images were taken at 0.5, 1, 2, 6, 24, and 48 h post-tail vein injection. Also, at 48 h, the major organs, such as the liver, spleen, kidneys, heart, lungs, and brain, were harvested and imaged *ex-vivo* (Figure 6B). Given the absence of tumor mass or any other diseased tissues, DPNs were observed to accumulate within the abdominal cavity over time (Figure 6A). The lack of fluorescence signal in the bladder during the experimental observations would confirm the stable association of the DSPE-Cy5-B molecules with the structure of DPNs. However, it should be highlighted that a moderate reduction in the Cy5 signal is observed in the abdominal cavity (Figure 6A), which would suggest a modest but sustained release of Cy5 from DPNs in agreement with the release studies of Figure 4B for the directly loaded molecule. The insets of Figure 6B confirm that the DPN accumulation in the liver. Indeed, this was expected as liver would be the natural site of particle accumulation given the lack of any diseased tissue.

## Conclusion

Two different loading strategies were documented to encapsulate hydrophobic and hydrophilic imaging and therapeutic compounds within the polymeric matrix of Discoidal Polymeric Nanoconstructs (DPNs). Two near-infrared imaging molecules were considered, namely Cy5-A and Cy5-B, together with two therapeutic molecules, methotrexate (MTX) and doxorubicin (DOX). The hydrophobicity/hydrophilicity ratio and molecular weight of these molecules were modulated by conjugating them directly with lipid (DSPE) and polymeric chains (PEG 1 kDa). In the “direct loading” method, the compounds were first dissolved in the polymeric paste forming the DPNs and then, together with this paste, applied to the PVA template. This approach suffered from the current sub-optimal fabrication yielding of DPNs returning encapsulation efficiencies lower than 1%. In the “absorption method,” the compounds were resuspended in water at high concentrations (1 mg/ml) and dragged inside the polymeric matrix of DPNs upon rehydration. This approach required a fine-tuning between the compound hydrophobicity and molecular weight and returned encapsulation efficiencies as high as 80%. Specifically, the highest encapsulation was documented for compounds with a moderate hydrophobicity and low molecular weights. These two features were also shown to affect the release profiles of the loaded compounds. In general, direct loading was associated with lower release rates as compared to absorption loading for a given compound. This was ascribed to the fact that the compounds in the absorption loading are mostly confined in the vicinity of the DPN surface and are therefore more rapidly released into the surrounding aqueous environment. Differently, in direct loading, the compounds are uniformly distributed within the polymeric matrix. The cytotoxicity properties of MTX and DOX loaded DPNs were tested on triple-negative breast cancer cells (MDA-MB-231). As expected, it was documented a delay in the cytotoxic activity *in vitro* mostly due to the hydrolyzation and release of the compounds from DPNs. Finally, in preliminary biodistribution studies, it was shown that direct loaded Cy5 compounds would stay firmly associated with DPNs during circulation and slowly leak out over an observational period of 48 h. Collectively, these results demonstrate that the pharmacological properties of DPNs can be finely tuned during the fabrication process by changing the loading strategies (direct vs. absorption) and compound properties (hydrophobicity and molecular weight). Future studies will focus more on further optimizing the loading and release conditions and pre-clinically demonstrate the therapeutic and imaging performance of this drug delivery platform in different disease models.

## Data Availability Statement

The datasets generated for this study are available on request to the corresponding author.

## Ethics Statement

All animal experiments were performed at the Italian Institute of Technology (IIT) animal facility according to the guidelines established by the European Communities Council Directive (Directive 2010/63/EU of 22 September 2010) and approved by the National Council on Animal Care of the Italian Ministry of Health. All efforts were made to minimize animal suffering and to use the minimal number of animals required to produce reliable results.

## Author Contributions

MF performed all the loading and release experiments, and fabricated the particles. IR fabricated and characterized the particles. AP fabricated the particles and performed the *in vivo* experiments. MB, VD, and MD performed all the cell viability studies. PD designed the experiments, wrote the manuscript, and oversaw all the research activities. All authors have discussed the results and read the manuscript.

### Conflict of Interest

The authors declare that the research was conducted in the absence of any commercial or financial relationships that could be construed as a potential conflict of interest.
