# Supplementary material for: Optimizing the Pharmacological Properties of Discoidal Polymeric Nanoconstructs Against Triple-Negative Breast Cancer Cells
Source: Front Bioeng Biotechnol. 2020 Feb 19;8:5. doi: 10.3389/fbioe.2020.00005 (PMC7042398; doi:10.3389/fbioe.2020.00005)
Supplement: Supplementary file 1 [file Data_Sheet_1.pdf]

SUPPORTING INFORMATION

OPTIMIZING THE PHARMACOLOGICAL PROPERTIES OF DISCOIDAL POLYMERIC NANOCONSTRUCTS AGAINST TRIPLE NEGATIVE BREAST CANCER CELLS

Miguel Ferreira[1], Ilaria Francesca Rizzuti[1,2], Anna Lisa Palange[1], Maria Grazia Barbato[1,2], Valentina Di Francesco[1,2], Martina Di Francesco[1], and Paolo Decuzzi[1]*

[1] Laboratory of Nanotechnology for Precision Medicine, Fondazione Istituto Italiano di Tecnologia, Via Morego 30, Genoa 16163, Italy

[2] Department of Informatics, Bioengineering, Robotics and System Engineering, University of Genoa, Via Opera Pia, 13 Genoa 16145, Italy

♣ Corresponding author: Paolo Decuzzi Ph.D., paolo.decuzzi@iit.it

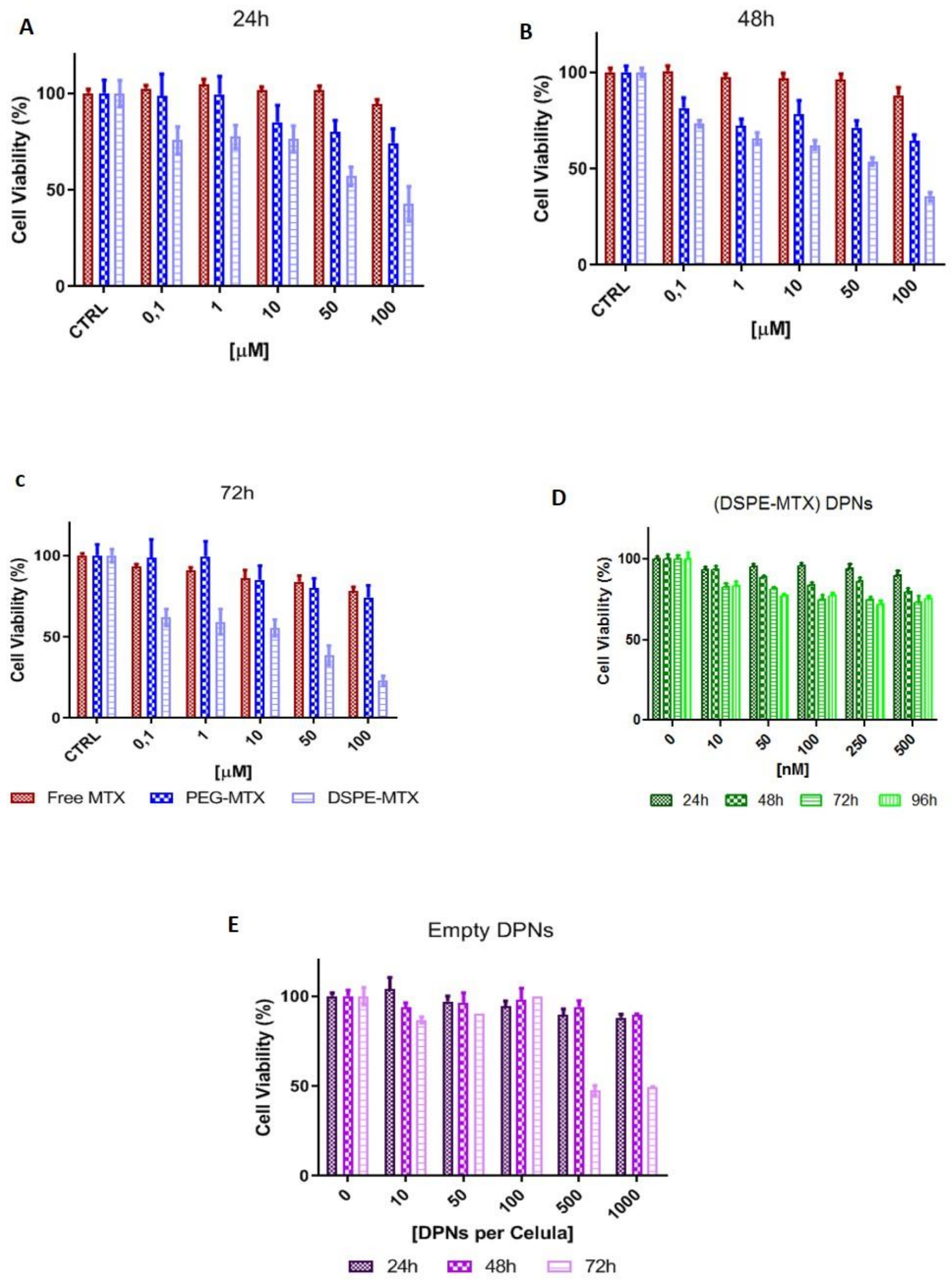

Supplementary Figure. 1. Cytotoxic activity of MTX prodrugs and MTX-loaded DPNs A. Cell viability analysis of **A.** free-MTX; **B.** PEG-MTX; and **C.** DSPE-MTX at 24, 48, and 72 post-incubation with MDA-MB-231. **D.** Cell viability of DSPE-MTX DPNs at 24, 48, 72 and 96h post-incubation with MDA-MB-231. **E.** Cell viability of empty DPNs at 24, 48, and 72 h post-incubation with MDA-MB-231.

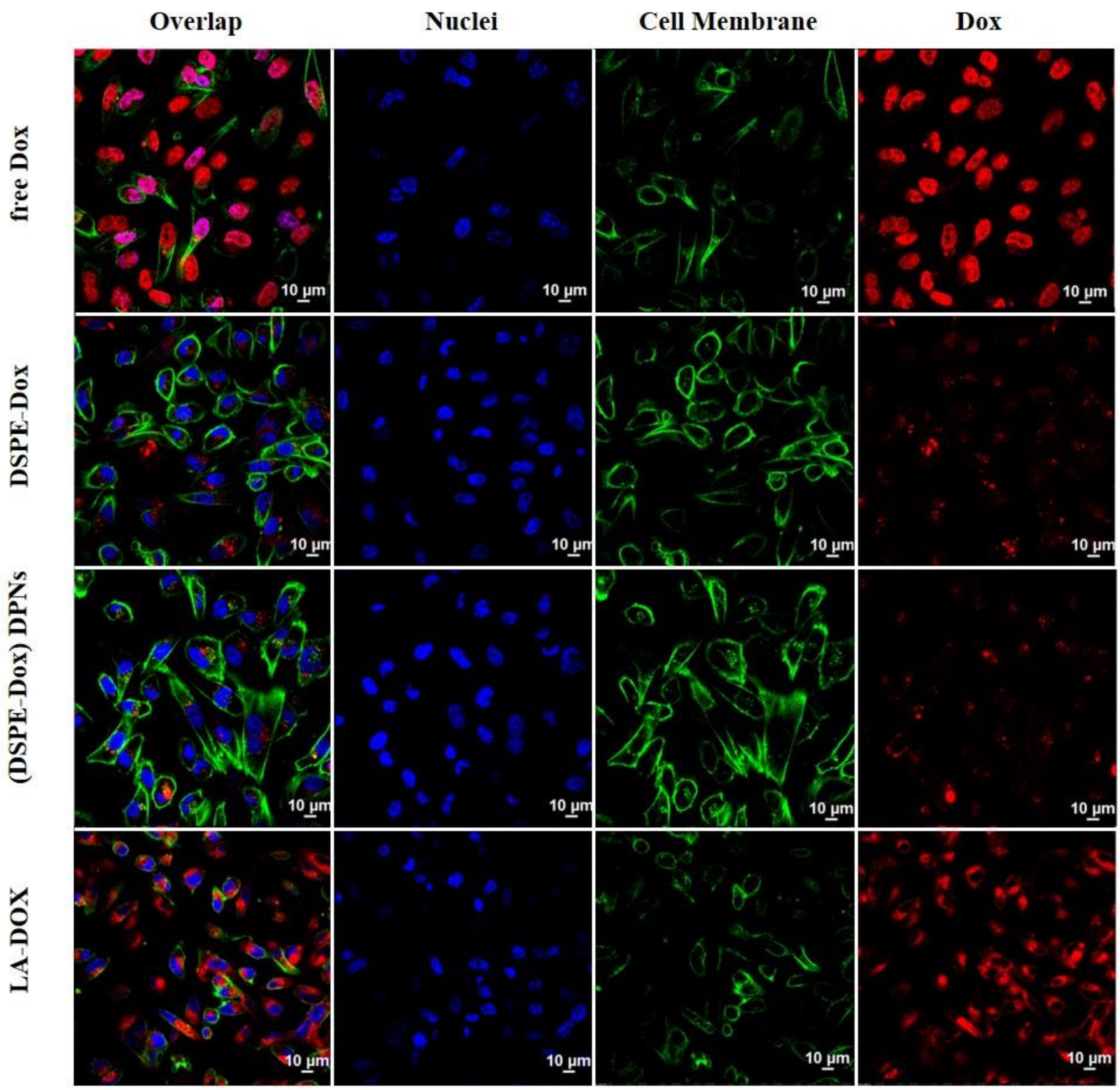

Supplementary Figure.2. Confocal microscopy analysis of free-DOX, DSPE-DOX, (DSPE-DOX)DPNs and LA-DOX at 24h post-incubation with MDA-MB-231.

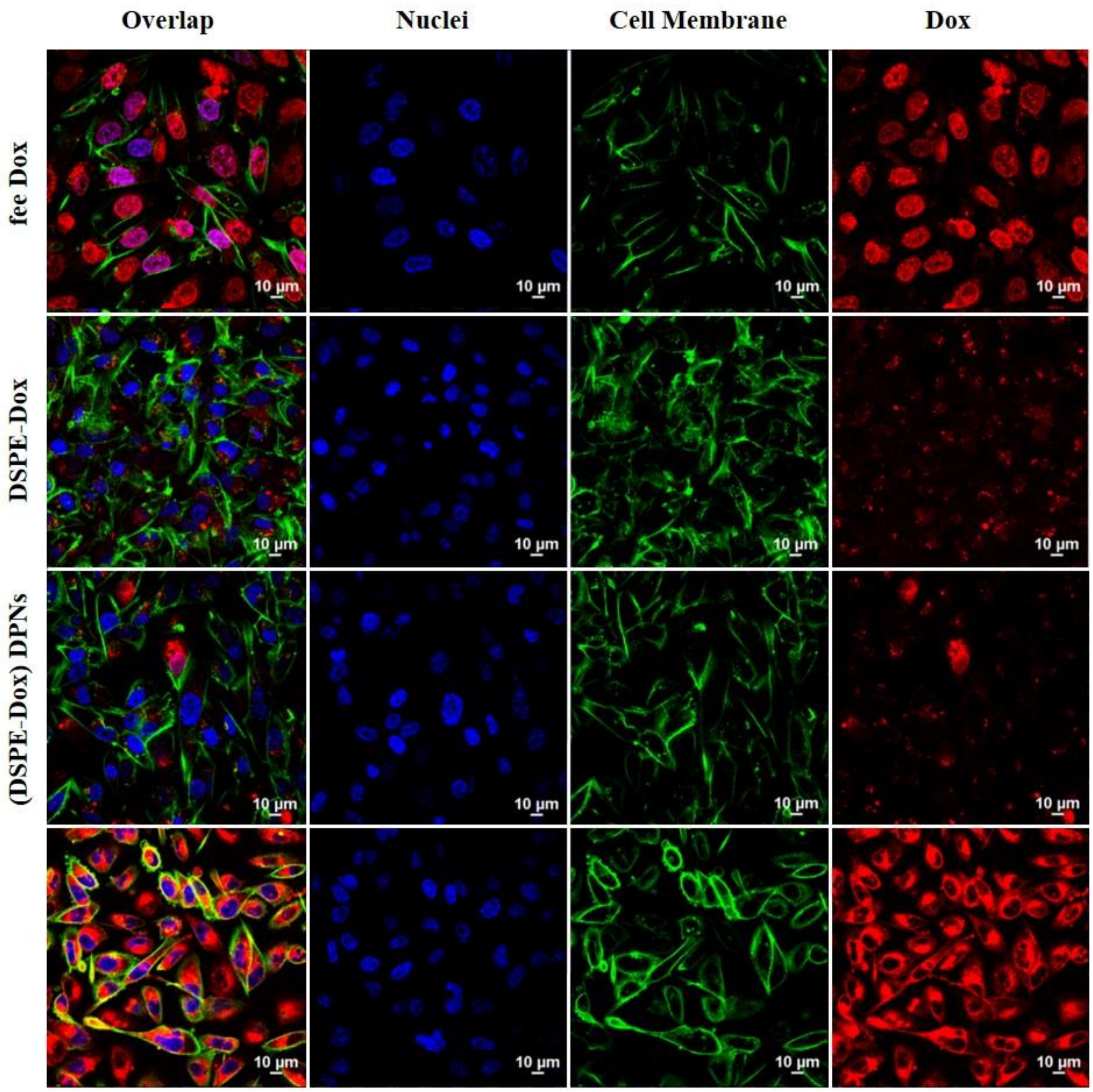

Supplementary Figure.3. Confocal microscopy analysis of free-DOX, DSPE-DOX, (DSPE-DOX)DPNs and LA-DOX at 48h post-incubation with MDA-MB-231.

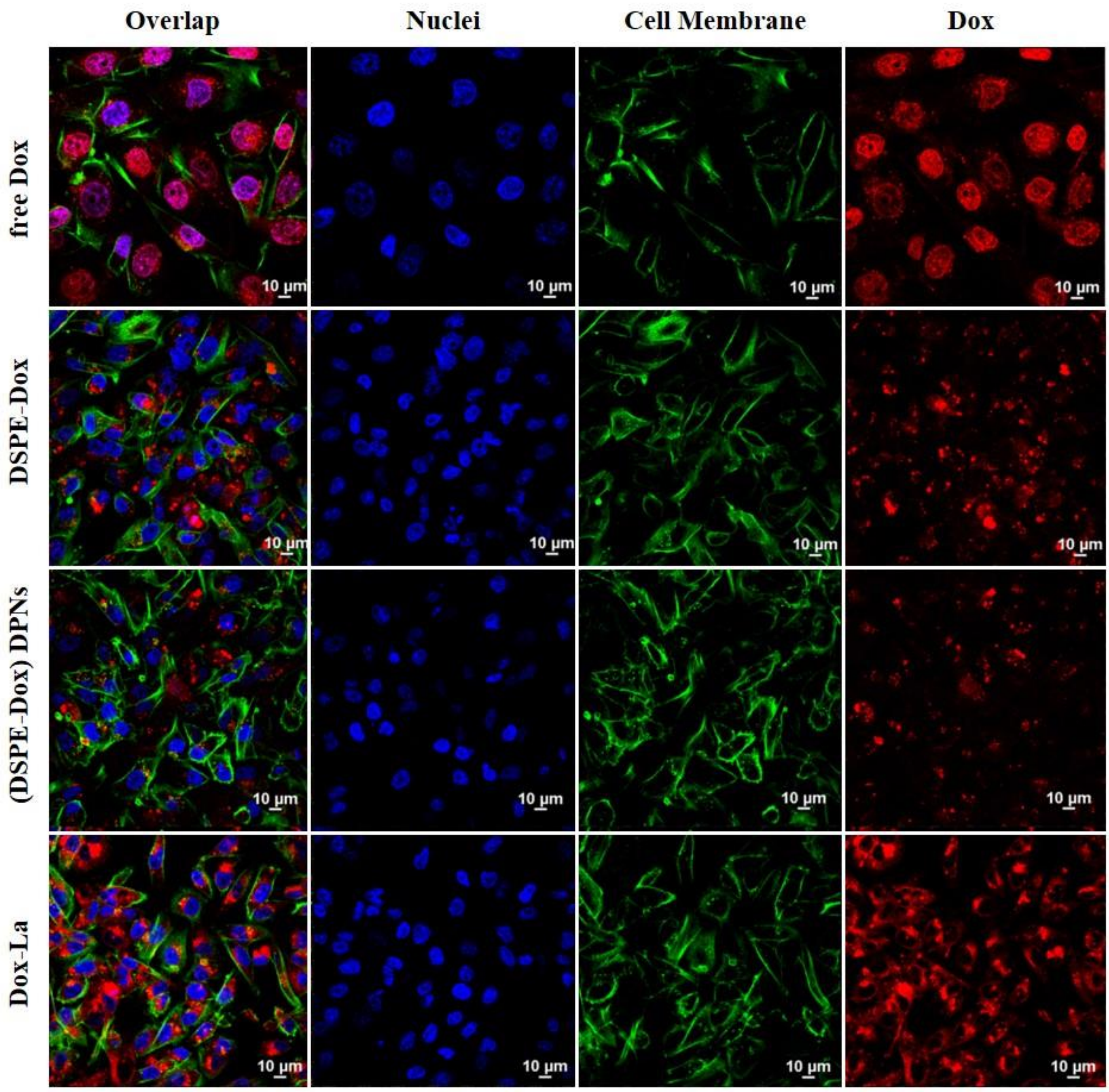

Supplementary Figure.4. Confocal microscopy analysis of free-DOX, DSPE-DOX, (DSPE-DOX)DPNs and LA-DOX at 72h post-incubation with MDA-MB-231.

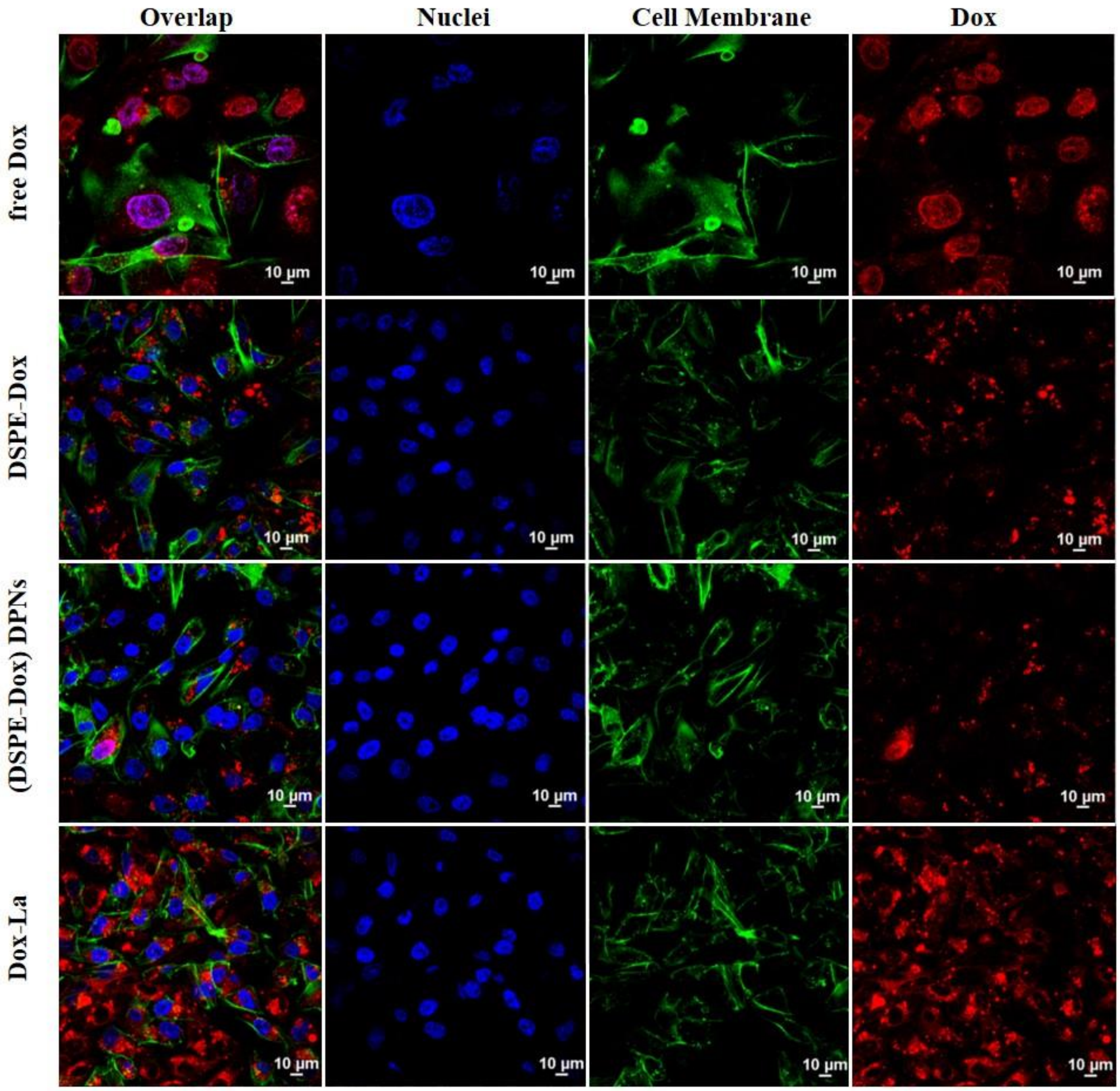

Supplementary Figure.5. Confocal microscopy analysis of free-DOX, DSPE-DOX, (DSPE-DOX)DPNs and LA-DOX at 96h post-incubation with MDA-MB-231.
